# Eyecare at Tilganga Institute of Ophthalmology during COVID–19 pandemic

**Published:** 2020-09-01

**Authors:** Reeta Gurung

**Affiliations:** 1 CEO: Tilganga Institute of Ophthalmology, Kathmandu Nepal.

**Covid-19 is having profound repercussions on eye care service delivery across the world. Hospitals in low- or middle-income countries need to adapt quickly to prevent its spread. We share some of our learnings and challenges to help others cope with this pandemic.**

**Figure:**
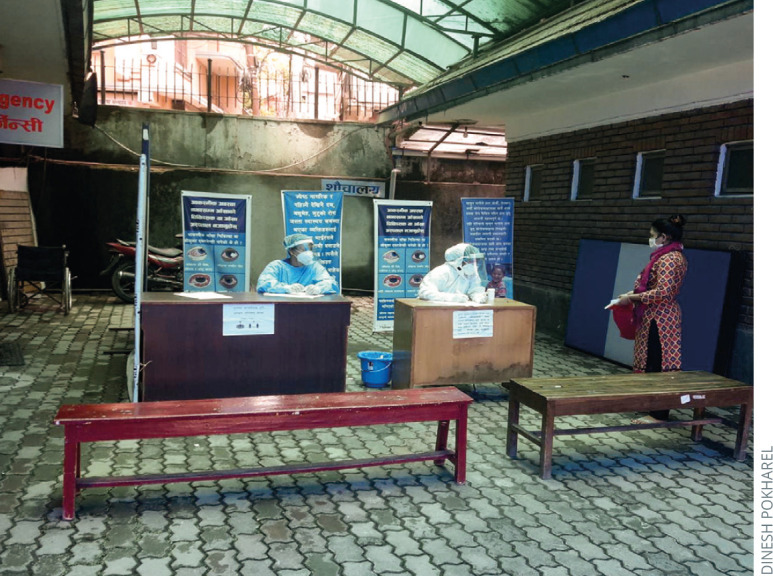
A patient entering the triage area at Tilganga Institute of Ophthalmology. **NEPAL**

The COVID-19 pandemic has had profound repercussions for eye care delivery at all hospitals in Nepal, including the Tilganga Institute of Ophthalmology (TIO) in Kathmandu.

Nepal reported its first case on 13 January 2020 and the second case on 23 March, and the government announced a complete lockdown the following day.

The ten-week gap between the first and second case gave us time to prepare for the pandemic. We started taking the following preventive measures – to ensure safety of our staff and patients – before Nepal went into a complete lockdown.

- We set up a triage area at the hospital and now use a thermal gun to measure the temperature of all patients and staff members as they come in; doing so helps us to manage patients who come to the hospital without appointments (fewer than a hundred per day).
- We segregate patients depending on their travel history and symptoms and see patients with a positive travel history and symptoms in a separate clinic, near the triage area. This avoids the need for these patients to visit the main outpatient department. A separate pharmacy is also set up for them.
- We prepare alcohol-based sanitiser in the hospital as per the World Health Organization’s guidelines. Everyone has to sanitise their hands before entering the hospital premises.
- We put up posters on Covid-19 prevention within the hospital. Some of the messages on our walls include: “Do not come to the hospital unless it is necessary,” “Maintain a physical distance of three feet,” “Please sanitise your hands here,” etc.
- We shared a mobile phone number with our patients via our website, local newspapers and online news websites so they could call us for advice. Although this was not a toll-free service, the consultation was free.
- We divided our medical and paramedical staff members into six groups. Each group comes to the hospital only once a week to minimise their exposure to asymptomatic COVID patients.
- All staff members coming in contact with patients wear full personal protective equipment (PPE), including face shields and N95 masks, all of which are made locally. We sanitise and sterilise the examining equipment and instruments after each use.
- We postponed all surgical procedures except for emergency services such as perforating injuries, infants with retinopathy of prematurity, oncology, emergency retinal surgery, and therapeutic grafts.

**Figure:**
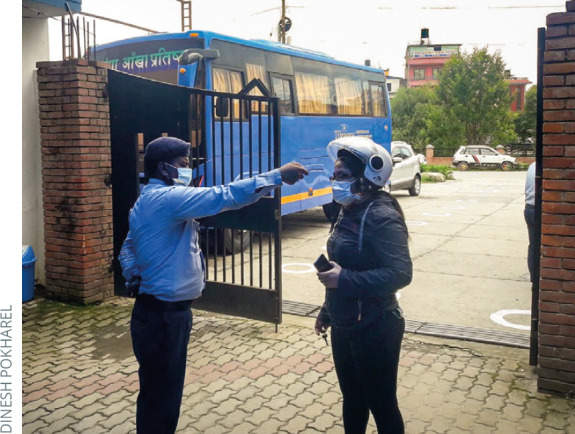
Staff member at TIO having her temperature measured with thermal gun. **NEPAL**

## Some of the challenges we continue to face during the lockdown are

- Keeping staff members safe
- Motivating personnel to come and work in the current situation
- Keeping the public safe
- Financial viability. Ensuring that the institution can function without patients is our biggest concern. While we pay personnel their basic salaries, extra perks are on hold for the time being. All budgets and expenses related to staff training, travel, and hospitality are also on hold.

## Lessons learnt

- **Preparedness.** Preparation is the key to get over the crisis. We were in a better position than our peers in other countries because we had more time to prepare ourselves.
- **Morale.** To keep the morale of the medical staff high, it was important that we looked after them well. This included providing personal protective equipment, transport to work, and food.
- **Long-term financial viability.** We started financial prudence measures as soon as possible.
- **Regular communication.** The board, management and staff members communicated regularly.

